# Portuguese Medical Students’ Interest for Science and Research Declines after Freshman Year

**DOI:** 10.3390/healthcare9101357

**Published:** 2021-10-12

**Authors:** Margarida Pereira, Gustavo Correia, Milton Severo, Ana Cristina Veríssimo, Laura Ribeiro

**Affiliations:** 1 Department of Public Health and Forensic Sciences, and Medical Education, Faculty of Medicine, University of Porto, 4200-319 Porto, Portugal; mmiguel06@gmail.com (M.P.); g_b_correia@hotmail.com (G.C.); averissimo@med.up.pt (A.C.V.); 2 Institute of Biomedical Sciences Abel Salazar, University of Porto, 4050-313 Porto, Portugal; milton@ispup.up.pt; 3 Institute of Public Health, University of Porto, 4050-091 Porto, Portugal; 4 I3S—Instituto de Investigação e Inovação em Saúde, Universidade do Porto, 4200-135 Porto, Portugal

**Keywords:** medical education, undergraduate research, physician-scientists, scientific skills, medical students

## Abstract

The integration of scientific research into medical curricula remains insufficient despite its advantages for medical students’ professional development and the advancement of medicine. This study aimed to evaluate the impact of first-year medical course attendance on medical students’ attitudes and perceptions towards scientific research and clinical practice, while also assessing the contribution of sociodemographic and academic factors. Two hundred and thirteen medical students self-administrated a questionnaire at the beginning and at the end of the first school year. Their responses were compared and two regression models were calculated to assess factors influencing students’ attitudes and perceptions. After freshman year, students displayed significantly lower positive attitudes towards science and research. Their motivation to perform research and to integrate it into the curriculum also decreased, while the importance attributed to research skills for clinical practice increased. Motivation to perform research and negative attitudes were positively and negatively associated with grade point average (GPA), respectively. Female students and those who attended public secondary schools attributed greater importance to communication skills. This study reinforces the need to early develop research skills and positive attitudes in medical students, motivating them to become physician-scientists. Additional follow-up studies may offer further contributions to the integration of research into medical curricula.

## 1. Introduction

The integration of scientific research-related activities into medical curricula can improve the quality of health care systems by fostering the interest of future physicians in developing research alongside clinical practice, thereby increasing the number of physician-scientists, who are instrumental in this process of translational research [1,2,3]. However, such integration remains insufficient [4,5,6,7].

The number of physicians [8,9,10] and medical students [9,11,12] interested in scientific research has declined, although a scientific education is needed to help developing scientifically-minded physicians [13,14,15]. The participation of medical students in research activities improves their scientific thinking [16], critical appraisal and problem-solving skills, key for best clinical practice [9,17,18,19]. Additionally, it contributes to better informed career choices and improved knowledge and attitudes towards research, while being associated with higher scientific productivity [4,20].

Due to the expensive costs [8,11,21], time [18,21] and intellectual demands of scientific research [21,22,23], physicians find it very difficult to reconcile research with medical practice. For medical students, the absence of a research role-model or mentorship [8,18,24], the lack of meaningful research experiences [10,21], adequate skills [23] and teaching on basic scientific principles [5,24], and inflexible curricula [4] are other obstacles to scientific research.

Therefore, from an early stage, medical students must have a broad scientific training [9,25,26]. Participating in mentored research projects as undergraduate medical students is suggested to be the best option to develop and improve research skills [13,19,27]. Other strategies may include compulsory graduation theses and allowing for intercalated BSc degrees [4,14,28]. Publishing [21] and attending conferences [9,29,30] allows students to mold their professional attitude towards clinical practice and to integrate scientific communities. Mentored research projects also stimulate autonomy [13], by promoting self-directed learning, leadership, creativity [27], time management, critical [18] and communication skills [9,31] and excellence in clinical practice [21].

Many prominent European [28,32,33,34] and North American [9,35,36] medical schools have integrated compulsory undergraduate research activities into their medical curricula. In 2007, alongside the Bologna Process, an attempt was made to properly integrate scientific research into Portuguese medical schools’ curricula [37,38]. Consequently, medical students may further develop a broader range of differentiated scientific skills [31].

Positive attitudes towards science and scientific research are critical for medical education [6]. Its early stimulation enhances students’ development and critical appraisal and assessment skills through the learning of evidence-based medicine [6,39].

Despite the appointed benefits, more studies around attitudes and perceptions towards scientific research are needed [6,20,40], especially on how they are affected and how they can be modified [6,39].

As undergraduates, and for a limited period [39], medical students who participate in minor mandatory scientific methodology courses during the second year have their positive attitudes towards science increased [6,39]. According to previous studies, this increase is not related with medical students’ attitudes towards science and scientific research at the beginning of medical school, and only longstanding programs would be able to effectively change attitudes towards science [39,41].

Evidence suggests that medical students’ attitudes towards science and scientific research are affected by participation in research projects [16,42,43], sex [10,31,44], academic year [31,35] and stimuli towards research [45]. In previous studies [7,31], perceptions and attitudes of medical students towards science and scientific research, as well as factors influencing them, have already been assessed in order to create a useful component to guide students in their professional development, limiting the breach between research and clinical practice [2,46], ultimately benefiting patients [19].

Attitudes are described as context dependent [39,47,48] and mostly fixed inclinations towards a positive or negative response to an idea [39,47], whereas perceptions are the processes of appreciation, understanding and response to a stimulus [49]. Evidence [31,40] suggests that perceptions about research are related to motivation for research.

A skill is something that can be learned and mastered [21]. At this level, research skills are the ability to search for knowledge, and even best practices [13,27,29]. Organization skills are the ability to best use time and effort in all aspects of life [8], and communication skills are the ability to communicate with patients [50], including verbal and non-verbal elements [51], and research outcomes [52].

In a cross-sectional study [31], we observed that medical students seem to lose interest for science and research halfway through medical course when compared to the beginning. In this study, we sought to assess whether that decline in medical students’ interest for scientific research might occur at an even earlier stage of medical training.

### Research Objectives

The primary goal of this follow-up study was to evaluate the impact of first year medical course attendance on the attitudes and perceptions towards scientific research and clinical practice of medical students. As a secondary goal, it was assessed if factors such as age, sex, type of secondary school attendance, place of residence, previous participation in research projects and grade point average (GPA) influenced students’ attitudes and perceptions towards scientific research and clinical practice.

## 2. Materials and Methods

### 2.1. Study Design and Participants

This follow-up study involved all medical students enrolled in the first year of a six-year medical course at the Faculty of Medicine of the University of Porto (FMUP). FMUP lectures the Integrated Master’s Degree in Medicine and is a cutting-edge organization, having one of the highest admission GPA of Portuguese undergraduate programs.

FMUP has no structured research programs incorporated directly into the undergraduate curriculum. During freshman year, medical students do not have extensive contact with scientific research, except for some basic science courses (e.g., Biochemistry, Molecular and Cellular Biology) where they engage in some lab tasks.

At the end of the first academic year, out of the 257 students enrolled in that first year, 213 (82.88%) self-administrated voluntarily and non-anonymously a paper version of the “Importance of Scientific Skills for Clinical Practice (ISS4CP)” questionnaire [31] and the informed consent form, which were handed to students after classes.

### 2.2. Survey Instrument

The ISS4CP questionnaire [31] comprised four domains: (I) General Information, including age, sex, type of secondary school attendance (public or private), place of residence (Porto or other) and previous participation in research projects; (II) Attitudes of medical students towards science and scientific research, consisting of Positive and Negative Attitudes and the willingness to integrate scientific research into the medical curriculum (Integration); (III) Motivation of medical students to perform research while in medical school (Motivation); and (IV) Perception of medical students on their scientific skills and its importance for clinical practice, encompassing communication, research and organization skills. Communication skills included writing, oral and visual communication; research skills included the ability to perform literature searching, to cope with information technology, to analyze data, and English proficiency; and organization skills included teamwork, time management, problem-solving and self-improvement in learning ability. The questionnaire has been priorly published and validated, revealing adequate reliability for all its dimensions (Cronbach’s alpha = 0.828–0.939) [31].

All Likert scales used, reversed for the purpose of this study, ranged from 1 (total agreement, very high, very important or good) to 4 (total disagreement, low, not important or bad). To calculate the score of each item, the average of the responses was computed and its analysis is straightforward.

### 2.3. Statistical Analysis

Comparisons were made between the answers of 234 medical students, at the beginning of the first year, the first moment, and the answers of 213 medical students, at the end of the first school year, the second moment, with a follow-up response rate of 91%.

Data had a normal distribution. Paired-samples t-tests were calculated to compare scores within each dimension and identify differences between medical students’ attitudes and perceptions towards science and scientific research in the two moments of data collection. Two linear regression models were computed to identify which factors were associated with the dimensions being studied: one adjusted for age, sex, type of secondary school attendance, place of residence, previous participation in research projects and GPA of first year courses (Model 1), and another adjusted for all variables in the first model plus the dimension score at the first moment of data collection (Model 2). Statistical analysis was performed using R 2.12.1. [53] and statistical significance was defined at *p* < 0.05.

### 2.4. Ethical Approval

Ethical principles followed the frameworks approved by FMUP. The research was carried out in accordance with the Declaration of Helsinki.

All participants received written information about the background of the study and contact details of the person responsible for it. Participation in the study was voluntary. Data confidentiality was warranted during the whole research process.

## 3. Results

### 3.1. Descriptive Statistics

An analysis was conducted to compare the characteristics of the participants who responded to the ISS4CP questionnaire in both moments and those who missed the second moment. As shown in Table 1, there were no significant differences between participants and nonparticipants, except for GPA of all first-year curricular units, where nonparticipants had significantly lower GPA compared to participants.

### 3.2. Medical Students’ Attitudes and Perceptions towards Science and Scientific Research

As seen in Table 2, after attending the first year, students significantly decreased their positive attitudes towards science and scientific research, their willingness to integrate scientific research into the medical curriculum, and their motivation to perform research during medical school, despite attributing a greater importance to research skills for clinical practice.

The regression models analysis, presented in Table 3 and Table 4, show that in Model 1 the only statistically significant factor, affecting positive attitudes towards science and scientific research, was to reside in a place other than Porto (0.117, 95% CI: 0.020; 0.213). In Model 2, after adjustment for the score of that dimension in the first moment of data collection, having a place of residence other than Porto remained statistically significant (0.094, 95% CI: 0.008; 0.180). Additionally, negative attitudes towards science and motivation to do research while in medical school were, respectively, negatively (−0.019, 95% CI: −0.037; −0.0004) and positively (0.045, 95% CI: 0.009; 0.082) associated with the GPA of first year of medical course. Furthermore, in Model 2, the score of each dimension in the first moment of data collection was negatively associated with all dimensions in analysis: positive attitude (−0.474, 95% CI: −0.598; −0.350), negative attitudes (−0.462, 95% CI: −0.578; −0.347), willingness to integrate research into medical curriculum (−0.824, 95% CI: −0.975; −0.672), motivation to do research during medical school (−0.426, 95% CI: −0.552; −0.300), importance attributed to communication skills for clinical practice (−0.542, 95% CI: −0.664; −0.420), importance attributed to research skills (−0.560, 95% CI: −0.686; −0.434) and importance attributed to organizational skills for clinical practice (−0.589, 95% CI: −0.702; −0.475). The same model reveals that the importance attributed to communication skills for clinical practice by male medical students was lower when compared to female students (−0.129, 95% CI: −0.233; −0.024), and that students who attended private secondary schools also attributed a lower importance to communication skills when compared to those who attended public secondary schools (−0.044, 95% CI: −0.164; −0.076).

## 4. Discussion

Our results show a decrease in positive attitudes towards science and scientific research, willingness to integrate scientific research into the medical curriculum, and motivation to perform research during medical school after attending first year courses, although medical students attributed greater importance to research skills for clinical practice. In line with previous findings [31], where mid-course medical students scored lower in all aforementioned dimensions compared to their peers at the beginning of medical school, this study also points to a decline in medical students’ interest for science and research after entering medical school. Furthermore, current results suggest that such a decline in students’ interest for research seems to occur at an even earlier stage of medical training, even though here the importance attributed to the skills for clinical practice increased after students had attended the first year. These concerning observations might be related to students’ previous exposure to theory-based education, in both secondary and medical school, not keen on research training, aggravated by the lack of a structured program of scientific research for undergraduate medical students in our school, directing them towards clinical practice. Despite this seemingly insufficient contact with scientific research, it still appears to significantly raise students’ awareness of its importance for their professional development and even for clinical practice when compared to the first moment of evaluation, nonetheless leaving room for improvement and adjustments. Additionally, first year school activities at FMUP are not compatible with extracurricular activities and the demands of full scientific research experiences. The lack of a proper research model at an early stage of medical students’ professional development is also known to lead them to prioritize academic components other than research [21,31]. Still, as the first moment assessed was at the beginning of the year, prior to any contact with medical school program and the workload students of the course, medical students’ expectations might have had further importance when assessing those results [31,34,54]. At this level, stimulating positive perceptions of research, self-efficacy and curiosity have been found to positively influence motivation for research among first year medical students [26].

First year medical students with higher GPA had a greater motivation to perform research during medical school and lower negative attitudes towards science and scientific research. This seems to be in accordance with previous studies, where students who participated in undergraduate research projects, regardless of disciplinary field, had a higher GPA [25], and higher ranked medical students had more positive attitudes towards science [55].

Another worrying observation relates to the score of each dimension at the first moment of data collection being negatively associated with all the factors in analysis. Although medical students with the least positive attitudes towards science at the first moment now have more positive attitudes, students with the most negative attitudes have less negative attitudes towards science, students who least agreed with the integration of scientific research into the medical curriculum now agree more, the least motivated students are now more motivated to perform scientific research during medical school, and students who attributed the least importance to research skills for clinical practice now attribute greater importance to them; the reverse was also observed. Despite the success of first year medical curriculum in bringing medical students with the lowest scores closer to science and scientific research, the program was not able to sustain the high scores observed in some students at the first moment. Once again, as the first moment of assessment was at the beginning of the year, the lack of a proper research model [21], the workload, expectations [54] and non-participation in significant research activities [6,31] might have influenced the scores at the second moment of data collection, despite students recognizing the importance of scientific skills for clinical practice [30,31]. Additional efforts to fully implement a scientific research program are needed to avoid compromising professional development of future doctors and the quality of health care systems. As resources are now limited and with Portuguese medical students exceeding need, it is time to support these students to attain differentiated scientific skills [31], overcoming their lack of scientific literacy.

As in this study, previous research [31] has already disclosed the influence of sex on the importance medical students attribute to communication skills for clinical practice.

The evaluation of the perceptions of the students about their education is a valid measure of medical education itself [56,57]. Self-perceived skills are a component of self-efficacy [3,31] and its evaluation reveals the motivation to improve scientific skills, identifying areas for improvement.

The finding that having a place of residence other than Porto affected positive attitudes towards science and scientific research motivates further work. Differences regarding place of residence and other characteristics of the first-year medical student population should be explored.

In contrast to previous findings [7,31], prior participation in research was not associated to any factor in analysis, most likely due to sample issues. In a meta-analysis [4], research engagement during medical school also displayed no significant correlation with the attitudes or motivation of the students towards research.

The global shortage of physician-scientists [8,9,58] compromises the medical workforce’s ability to sustain evidence-based practice through the understanding, critical appraisal and application of research [6,9,39], which is paramount to medical advancement, particularly of translational medicine, and to improve patient care [2,43]. Our results reinforce the need to further develop, from the beginning of future doctors’ preparation, research skills and positive attitudes that stimulate their interest in pursuing a scientific career. At this level, medical curricula need to be adjusted to integrate longstanding and well-structured research programs [26,39,41], ensuring an adequate balance between scientific and clinical preparation, while avoiding student overload and demotivation [8,21,33]. Exposure to research activities, such as projects or “Journal Club” sessions, that provide clear directions, learning objectives [32,33,36], authentic learning experiences, with opportunities for students to publish their work [40], and which emphasize the relevance of research for professional practice [21,26], underpinned by adequate role-models and mentorship [29,33,35] are advocated. Based on these actions, medical students are more likely to display positive attitudes [19,39], self-confidence [16,19], self-efficacy [26] and motivation to pursue research as practitioners [18,19,43].

Further studies exploring factors that affect medical students’ attitudes and perceptions towards research are also recommended. This can offer more in-depth knowledge on the subject, enabling medical schools to better guide their actions to integrate scientific research in their curricula and allure students to this area.

In this study, it is possible to identify the following limitations: the results are based on self-reports and all participants attend the same school. This might lead to overestimation and recall bias. Further studies should be conducted using samples from different years.

## 5. Conclusions

After attending first year courses, medical students’ positive attitudes towards science and scientific research, motivation to engage in research activities and to include research programs into the medical curriculum decreased. Place of residence and GPA were significantly associated with medical students’ attitudes and perceptions, respectively. In turn, the importance attributed to communication skills for clinical practice was affected by sex and type of secondary school attendance.

This study reinforces the need to develop, from the beginning of the medical course, research skills and positive attitudes in future doctors, in order to counter the current shortage of physician-scientists, which may compromise medical advancement, particularly of translational medicine, and patient care. Thus, medical curricula need to be adjusted to ensure an adequate balance between scientific and clinical preparation. Exposure to well-designed and mentored research activities that emphasize the relevance of research for professional practice can foster medical students’ attitudes, self-perceived ability and motivation for a research career.

Additional follow-up studies can provide more accurate information regarding the design and integration of a proper research program in medical curricula.

## Figures and Tables

**Table 1:** Comparison of General Information between nonparticipants and participants.

	Nonparticipants (*n* = 21)	Participants (*n* = 213)	*p*-Value
Age (M (SD))	18.15 (0.37)	19.16 (5.04)	0.372
GPA of first year courses	8.93 (4.01)	11.98 (1.96)	<0.01
	*n* (%)	*n* (%)	
Sex			
Female	13 (61.9)	139 (65.3)	0.759
Male	8 (38.1)	74 (34.7)	
Type of secondary school attendance			
Public	12 (66.7)	151 (71.2)	0.683
Private	6 (33.3)	61 (28.8)	
Previous participation in research projects			
No	15 (71.4)	153 (71.8)	0.969
Yes	6 (28.6)	60 (28.2)	
Place of residence			
Porto	6 (28.6)	99 (46.5)	0.115
Other	15 (71.4)	114 (53.5)	

**Table 2:** Comparison between medical students’ attitudes and perceptions towards science and scientific research in both moments of data collection.

	1st Moment	2nd Moment	*p*-Value
	M (SD)	M (SD)	
**Attitudes towards science and scientific research**			
Positives Attitudes	3.36 (0.32)	3.26 (0.33)	<0.001
Negatives Attitudes	1.99 (0.31)	2.06 (0.31)	0.001
Integration in the curriculum	3.77 (0.25)	3.63 (0.28)	<0.001
**Motivation**	2.76 (0.56)	2.47 (0.61)	<0.001
**Communication Skills**	3.45 (0.41)	3.48 (0.42)	0.219
**Research Skills**	3.30 (0.41)	3.37 (0.42)	0.045
**Organizational Skills**	3.52 (0.43)	3.49 (0.39)	0.362

**Table 3:** Regression models of association between Positive Attitudes, Negative Attitudes, Integration and Age, Sex, Type of secondary school attendance, Previous participation in research projects and GPA of first year of medical course.

	Positive Attitudes		Negative Attitudes		Integration	
	Model 1	Model 2	Model 1	Model 2	Model 1	Model 2
	β (CI 95%)	β (CI 95%)	β (CI 95%)	β (CI 95%)	β (CI 95%)	β (CI 95%)
Age	−0.006 (−0.014; 0.003)	−0.004 (−0.012; 0.004)	0.004 (−0.004; 0.012)	0.003 (−0.005; 0.010)	−0.002 (−0.012; 0.007)	−0.002 (−0.009; 0.006)
Sex						
Female	Ref.	Ref.	Ref.	Ref.	Ref.	Ref.
Male	0.006 (−0.086; 0.099)	0.028 (−0.054; 0.111)	0.006 (−0.081; 0.094)	−0.005 (−0.081; 0.071)	0.001 (−0.098; 0.101)	0.009 (−0.071; 0.089)
Type of secondary school attendance						
Public	Ref.	Ref.	Ref.	Ref.	Ref.	Ref.
Private	−0.060 (−0.166; 0.047)	−0.046 (−0.140; 0.049)	0.001 (−0.098; 0.100)	0.034 (−0.053; 0.120)	−0.067 (−0.181; 0.047)	0.002 (−0.090; 0.095)
Previous participation in research projects						
No	Ref.	Ref.	Ref.	Ref.	Ref.	Ref.
Yes	−0.009 (−0.106; 0.089)	−0.008 (−0.094; 0.078)	0.047 (−0.042; 0.137)	0.031 (−0.047; 0.110)	−0.031 (−0.135; 0.072)	0.024 (−0.060; 0.107)
Place of residence						
Porto	Ref.	Ref.	Ref.	Ref.	Ref.	Ref.
Other	0.117 (0.020; 0.213) *	0.094 (0.008; 0.180) *	0.022 (−0.069; 0.113)	0.056 (−0.024; 0.0135)	0.071 (−0.033; 0.174)	0.012 (−0.072; 0.095)
GPA of first year of medical course	0.004 (−0.019; 0.027)	0.000 (−0.021; 0.020)	−0.019 (−0.040; 0.002)	−0.019 (−0.037; −0.0004) *	0.007 (−0.018; 0.032)	0.005 (−0.015; 0.025)
Score at the first moment of data collection		−0.474 (−0.598; −0.350) *		−0.462 (−0.578; −0.347) *		−0.824 (−0.975; −0.672) *

Model 1: adjusted for Age, Sex, Type of secondary school attendance, Previous participation in research projects, Place of residence and GPA of first year of medical course. Model 2: adjusted for all variables in Model 1 plus the dimension score on the first moment of data collection. * Statistically significant.

**Table 4:** Regression models of association between Motivation, Communication Skills, Research Skills, Organizational Skills, Age, Sex, Type of secondary school attendance, Previous participation in research projects and GPA of first year of medical course.

	Motivation		Communication Skills		Research Skills		Organizational Skills	
	Model 1	Model 2	Model 1	Model 2	Model 1	Model 2	Model 1	Model 2
	β (CI 95%)	β (CI 95%)	β (CI 95%)	β (CI 95%)	β (CI 95%)	β (CI 95%)	β (CI 95%)	β (CI 95%)
Age	0.004 (−0.012; 0.020)	0.004 (−0.010; 0.019)	−0.017 (−0.019; 0.005)	−0.006 (−0.016; 0.004)	0.000 (−0.013; 0.012)	−0.004 (−0.015; 0.006)	0.000 (−0.012; 0.012)	−0.001 (−0.011; 0.009)
Sex								
Female	Ref.	Ref.	Ref.	Ref.	Ref.	Ref.	Ref.	Ref.
Male	0.010 (−0.154; 0.173)	0.014 (−0.135; 0.163)	−0.055 (−0.0175; 0.066)	−0.129 (−0.233; −0.024)	−0.042 (−0.168; 0.083)	0.011 (−0.097; 0.119)	−0.001 (−0.126; 0.124)	−0.042 (−0.144; 0.060)
Type of secondary school attendance								
Public	Ref.	Ref.	Ref.	Ref.	Ref.	Ref.	Ref.	Ref.
Private	−0.151 (−0.338; 0.037)	−0.125 (−0.295; 0.046)	−0.100 (−0.239; 0.039)	−0.044 (−0.164; −0.076)	−0.125 (−0.270; 0.019)	−0.032 (−0.157; 0.093)	−0.045 (−0.188; 0.098)	−0.006 (−0.123; 0.111)
Previous participation in research projects								
No	Ref.	Ref.	Ref.	Ref.	Ref.	Ref.	Ref.	Ref.
Yes	0.010 (−0.181; 0.162)	0.054 (−0.102; 0.211)	−0.052 (−0.177; 0.074)	0.009 (−0.100; 0.118)	−0.080 (−0.212; 0.051)	−0.005 (−0.119; 0.108)	−0.119 (−0.248; 0.011)	−0.049 (−0.155; 0.058)
Place of residence								
Porto	Ref.	Ref.	Ref.	Ref.	Ref.	Ref.	Ref.	Ref.
Other	0.168 (−0.001; 0.338)	0.099 (−0.056; 0.254)	−0.039 (−0.165; 0.087)	−0.031 (−0.139; 0.077)	0.057 (−0.073; 0.188)	0.053 (−0.058; 0.165)	−0.090 (−0.218; 0.039)	−0.026 (−0.132; 0.080)
GPA of first year of medical course	0.039 (−0.001; 0.079)	0.045 (0.009; 0.082) *	0.010 (−0.020; 0.040)	0.000 (−0.026; 0.026)	0.028 (−0.003; 0.060)	0.017 (−0.010; 0.044)	0.026 (−0.005; 0.057)	0.014 (−0.012; 0.039)
Score at the first moment of data collection		−0.426 (−0.552; −0.300) *		−0.542 (−0.664; −0.420) *		−0.560 (−0.686; −0.434) *		−0.589 (−0.702; −0.475) *

Model 1: adjusted for Age, Sex, Type of secondary school attendance, Previous participation in research projects, Place of residence and GPA of first year of medical course. Model 2: adjusted for all variables in Model 1 plus the dimension score on the first moment of data collection. * Statistically significant.

## Data Availability

The data presented in this study are available on request from the corresponding author.
